# Body Composition and Dietary Intake of Combat Sports Athletes: A Systematic Review

**DOI:** 10.3390/nu18060884

**Published:** 2026-03-10

**Authors:** José Francisco Herrero Barceló, José Miguel Martínez Sanz, Mónica Castillo Martínez

**Affiliations:** 1 Nursing Department, Faculty of Health Sciences, University of Alicante, 03690 Alicante, Spain; jfhb1@alu.ua.es (J.F.H.B.); monica.castillo@ua.es (M.C.M.); 2 Research Group on Applied Dietetics, Nutrition and Body Composition (DANUC), University of Alicante, 03690 Alicante, Spain

**Keywords:** combat sports, body composition, dietary intake, nutritional recommendations, rapid weight loss

## Abstract

**Background/Objectives**: Combat sports are characterised by successive high-intensity and short-duration episodes (rounds) interspersed with short rest periods (intermittent nature). Athletes’ body composition and dietary intake are closely related to physiological demands, and they are determining factors in athletic performance. The aim of this systematic review was to describe the body composition, dietary intake, and food habits of male and female combat sports athletes, and to verify whether they met nutritional recommendations. **Methods**: A search was performed in the PubMed, Web of Science, and Scopus databases following the PRISMA statement. The timeframe for the search included studies from the year 2000 until 2 February 2026. Risk of bias was assessed using the STROBE and the Newcastle–Ottawa checklists. Initially, 328 documents were identified. The research focused on amateur, semi-professional, or professional athletes in boxing, karate, kickboxing, jiu-jitsu, taekwondo, judo, muay thai, and mixed martial arts (MMA). **Results**: After screening, 23 studies met the inclusion criteria and were included in the review. Most of the athletes, both men and women, had normal body mass indices (BMIs), with low or normal fat percentages and adequate muscle mass during both reference and pre-competitive periods. Regarding dietary intake, most of the athletes, male and female, had energy and carbohydrate intakes below official recommendations. Energy and nutrient intake decreased during pre-competition periods as a strategy for achieving pre-competitive rapid weight loss, which mainly occurred at the expense of lean mass. **Conclusions**: Despite maintaining adequate body composition, combat sports athletes reported an inadequate dietary pattern, especially during pre-competitive periods, which may negatively affect athletic performance.

## 1. Introduction

Combat sports are a sporting practice that encompasses multiple disciplines characterised by encounters in which two competitors attempt to physically confront and defeat each other [1]. Some of these sports are well represented in the Olympic Games (boxing, judo, wrestling, and taekwondo) [2]. Combat sports can be classified into three groups: grappling sports, striking sports, and mixed martial arts. In grappling sports (e.g., judo, jiu-jitsu, and wrestling), the objective is to achieve a dominant position on the ground or a submission through holds, throws, ground fighting, chokeholds, and joint locks [1,3]. In striking sports, competitors strike each other with their limbs to earn points or achieve a knockout (KO). Striking sports involve punching (boxing), kicking (taekwondo), and combinations of punching and kicking (kickboxing). Finally, mixed-style sports, such as mixed martial arts (MMA), involve a combination of wrestling and striking, making them physiologically more complex and requiring a wide range of technical skills [4]. Combat sports require a diverse physical and physiological profile adapted to each discipline in order to be successful in competition.

Combat sports are characterised by successive episodes of high intensity and short duration (rounds) interspersed with breaks lasting from seconds to minutes depending on the modality, and both the rules and the conditions for victory vary from one sport to another [5]. The intermittent nature of combat sports (repeated high-intensity efforts interspersed with lower-intensity actions) involves a high degree of aerobic metabolism; however, the high-intensity component also plays an important role in anaerobic metabolism [3]. Due to these physiological demands, carbohydrates are the primary energy substrate for this type of sport, so an adequate and sufficient supply of energy and carbohydrates is crucial to maximise glycogen reserves and increase the ability to maintain exercise [6].

Athletes’ dietary intake is a key factor to consider, as it is closely related to the physiological demands of the sport [7]. It is therefore essential to ensure proper energy and nutritional intake in order to reduce the risk of injury and avoid performance declines [8] and also a crucial tool for modulating the body composition of athletes [9]. Body composition is another determining factor in athletic performance and competition results [10]. It is important to mention that combat sports are classified by weight categories, with the aim of reducing disparities between fighters and promoting fair competition [11]. Most athletes tend to aspire to compete in weight categories below their usual weight to gain an advantage over their opponent, for which they often resort to acute weight loss (weight cutting) [11,12,13]. Acute weight loss consists of a rapid reduction in body mass, around 5%, in a short period of time (usually one week or less) [14]. Although the effects of acute weight loss on athletic performance are still controversial [15,16,17], the impact on competitors’ health is clearly negative, potentially causing serious physical and psychological damage [14,18]. Therefore, it is essential to provide safe, evidence-based dietary protocols that help reduce weight gradually to prioritise the athlete’s health and safety [6]. Nutritional strategies that promote gradual, long-term weight loss from body fat are recommended, and rapid weight loss strategies should be avoided, as they can lead to depletion of glycogen stores, dehydration, electrolyte imbalances, fatigue, injuries, increased heart rate, etc. [19]. The effectiveness of diet for weight management depends on three elements: distribution of energy intake in line with energy expenditure, macronutrient composition after training sessions (intake rich in protein and carbohydrates), and moderate calorie restrictions only in meals away from training sessions to avoid a decline in performance and ensure proper muscle recovery [20]. In addition, it can also be very useful to rely on the use of sports supplements to influence body composition and other factors that determine performance [21]. To ensure the appropriateness of nutritional protocols and to establish suitable weight-loss strategies, it is necessary to assess the body composition of combat sports athletes at different time points throughout the season.

There are several methods by which body composition can be determined, including anthropometry, bioelectrical impedance analysis (BIA), and dual-energy X-ray absorptiometry (DXA). Anthropometry refers to the set of measurements taken of the size and proportions of the human body. These procedures are standardised by the International Society for the Advancement of Kinanthropometry (ISAK) [22]. BIA is a non-invasive method that allows for body water, fat mass, and fat-free mass to be determined, based on the principle that electrical conductivity varies between different body compartments [23]. Finally, DXA is a method that allows for the determination of muscle mass, fat mass, and bone mineral density (BMD) through photon attenuation [24]. DXA is highly accurate and is considered the gold standard for measuring body composition [24].

Therefore, based on the above, it is essential to assess the body composition and dietary intake of combat athletes in order to optimise athletic performance and ensure good health, but research on nutrition and performance in weight-category sports remains limited by insufficient sex-specific evidence and significant methodological heterogeneity. Most studies have focused on male athletes, leading to the unjustified generalisation of results to females despite established sex-based physiological and hormonal differences [25,26]. This imbalance undermines the validity and applicability of current recommendations, particularly for female competitors. Moreover, inconsistencies in study design, dietary assessment, and control of confounding factors further restrict the comparability of findings [27,28]. Therefore, a systematic review is warranted to critically evaluate existing evidence, address sex disparities, and establish a more rigorous foundation for evidence-based nutritional guidance in weight-category sports.

Given these limitations, the aim of this systematic review was to describe the body composition, dietary intake, and eating habits of male and female combat athletes and to verify whether they complied with dietary and nutritional recommendations. Consequently, the initial hypotheses were as follows:

**Hypothesis** **1 (H1).** *There will be differences in various body composition variables among combat athletes depending on sex, competition level, and season phase.*

**Hypothesis** **2 (H2).** *There will be differences in dietary intake among combat athletes depending on sex, competition level, and season phase.*

## 2. Materials and Methods

### 2.1. Study Design

This systematic review based on existing scientific knowledge will be conducted to describe the characteristic body composition and dietary intake in combat athletes, both male and female. The PRISMA statement guidelines for the publication of systematic reviews were adopted [29] (see Supplementary Material—Tables S1 and S2). The protocol has been prospectively registered in the PROSPERO international prospective register of systematic reviews (registration number: CRD420251208258).

### 2.2. Eligibility Criteria

The Population, Intervention, Comparison, and Outcome (PICO) criteria for study inclusion and exclusion are shown in Table 1.

Regarding study design, this review includes both observational studies and intervention studies, provided they report relevant baseline or outcome data on either body composition, dietary intake, or both. Studies reporting on only one of these primary outcomes were intentionally included to ensure a comprehensive evaluation. This dual approach was necessary to independently verify the athletes’ physical status and their nutritional habits, ultimately allowing for a contrasted analysis of both domains. No limits will be applied regarding language, or publication status (pre-print, post-print, first online, or final) during the search phase, in order to maximise the sensitivity of the strategy. On the other hand, the exclusion criteria consisted of (i) subjects who were children (aged <12 years), older adults (aged >65 years), or injured or ill athletes; (ii) studies that did not specify measurement methods; and (iii) reviews, editorials, letters, and theses.

### 2.3. Data Sources and Search Strategy

The electronic databases consulted to obtain the most up-to-date information were PubMed, Web of Science (WOS), and Scopus. To find the largest number of articles related to the research aim, the words used in the search strategy were established considering the following: (i) combat sports; (ii) dietary intake; (iii) body composition; (iv) MeSH terms; (v) other terms described in MeSH as “synonyms” or free text terms; and (vi) the terms [Title/Abstract] attached to the “synonyms” or MeSH, which allow for the location of these terms in the title and abstract of the articles. For PubMed, a detailed strategy was constructed and adapted to the syntax of each database using the Polyglot Search Translator tool from the Systematic Review Accelerator platform [30]. The timeframe for the search included studies from the year 2000 until 2 February 2026, to ensure the inclusion of literature published after the establishment of the foundational evidence-based nutritional recommendations [31] and the subsequent implementation of structural safety regulations concerning rapid weight loss (RWL) and mandatory body composition assessment in weight-category sports [32]. Additionally, manual backward citation searches were conducted for all included studies. The search strategy is shown in Table 2 and the search was carried out in 2 February 2026.

### 2.4. Study Selection and Article Management

The selection of studies was carried out in two phases. In the first phase, the titles and abstract were analysed to identify studies that were eligible following selection criteria by 2 authors (J.F.H.B. and M.C.M). During the identification and screening process, a third researcher was consulted (J.M.M.-S.) to resolve discrepancies regarding the inclusion or exclusion of specific documents. The studies selected in the first phase underwent a second screening in which the full text was evaluated by the same authors independently and in duplicate. Finally, those studies that met the eligibility requirements proceeded to the data analysis and extraction phase.

### 2.5. Data Extraction

A table was created to summarise the characteristics and findings of the studies included in the review. The data extraction protocol consisted of the following variables:

- Study: Author and year of publication.
- Study design: Type of study (e.g., cross-sectional, cohort, case–control, randomised controlled trial, etc.).
- Objective: Purpose for which the study was conducted.
- Description of the sample: Number of subjects, sport discipline, and sex.
- Analysis/intervention: Analytical method used to compare descriptive variables or the defined and implemented experimental protocol.
- Main outcomes: Main findings relating to nutritional intake and body composition.
- Conclusions: Concise summary of the relevant results of the study.
- Measurement instruments: Methods, protocols, and tools used to collect body composition and dietary intake data.
- Season phase: Included to differentiate values collected between different cycles of a natural season (if specified). “Reference period” means that the data were collected at times far removed from any competition. “Pre-competition period” means that the data were collected within one week prior to the competition.
- Body composition: Characteristics and values of body composition by different methods.
- Energy intake: Kcal consumed expressed in Kcal/day and Kcal/kg/day.
- Macronutrients intake: Carbohydrate and protein consumed, expressed in g/day and g/kg/day; fat (lipids) consumed, expressed in g/day or % diet.
- Compliance with dietary intake recommendations: Degree of compliance according to reference sport nutrition scientific institutions (International Society of Sports Nutrition AND American Academy of Nutrition and Dietetics).

### 2.6. Risk of Bias (Study Quality and Data Collection)

The quality of the studies was independently assessed by two researchers (J.F.H.B. and M.C.M.) using the Strengthening the Reporting of Observational Studies in Epidemiology (STROBE) guidelines [33] and the Newcastle–Ottawa scale (NOS) [34,35]. A third reviewer was consulted to resolve discrepancies (J.M.M.S.) (see Tables S3–S5).

For cohort studies, the original version of the NOS [34] was applied, which uses a nine-star scoring system to judge each study in three main areas: (i) selection of study groups, (ii) comparability of groups, and (iii) determination of outcome. Studies were classified as having a low (7–9 stars), moderate (4–6), or high (0–3) risk of bias.

For cross-sectional studies, the adapted versions NOS-xs for analytical cross-sectional studies and NOS-xs2 for descriptive studies were used [35]. Both scales adapt the items to the particularities of each type of design. The NOS-xs maintains the nine-star scoring system, while the NOS-xs2 scores up to 4 stars: low risk of bias (3–4 stars), moderate (2), or high (0–1).

## 3. Results

### 3.1. Study Selection

**Figure 1:**
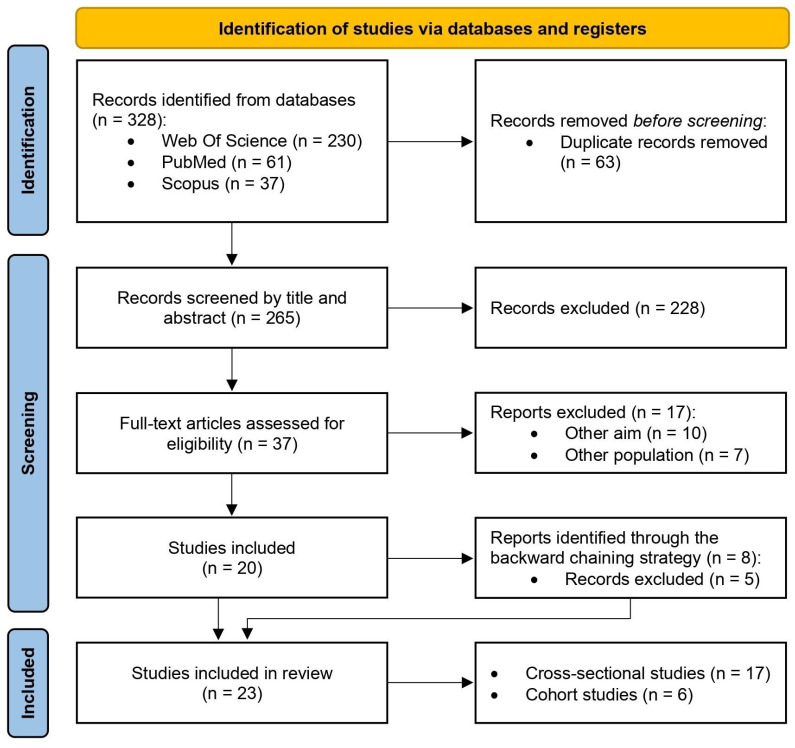
PRISMA flowchart.

A total of 328 documents were identified (230 in Web of Science, 61 in PubMed, and 37 in Scopus). After removing duplicates, 265 documents were retained. An initial screening was performed based on the title and abstract, and 37 documents were selected for full-text evaluation. Following a thorough analysis of the complete text, a total of 20 studies were chosen for inclusion in the review. The backward citation chaining method was used to gather as many potentially relevant articles as possible. Finally, a total of 23 studies were included in the systematic review. Figure 1 illustrates the article selection process.

### 3.2. Characteristics and Results of the Included Studies

The characteristics of the included studies are shown in Table 3. Most of the studies included in this review used a cross-sectional design [36,37,38,39,40,41,42,43,44,45,46,47,48,49,50,51,52], while six used a cohort design [53,54,55,56,57,58]. In general, the objective of the cross-sectional studies was to determine the body composition and dietary intake of combat athletes at a specific point in the season, while those that used a longitudinal design had more ambitious objectives, such as analysing changes in the body composition and dietary intake of athletes over different points in time, as well as establishing comparisons between reference periods and pre-competitive periods.

Out of the 23 studies included, six provided information on athletes from different combat disciplines [36,37,43,48,50,52]. However, most focused on a single combat sport: six articles focused on judo [42,45,51,53,56,58], three on jiu-jitsu [38,39,41], three on taekwondo [46,49,55], two on karate [44,47], one on boxing [57], one on kickboxing [40], and one on MMA [54]. Of these, only eight included both male and female samples [36,44,45,46,47,48,49,56]; in the rest of the studies, the sample involved only men, except for one, which included only female athletes [58].

Most studies, it was common to find that they used small sample, considering they focused on studying athletes belonging to a single sports club [38,39,41,42,44,45,49,53,54,55,56]. This trend was present regardless of the athletes’ sex and level of competition.

#### 3.2.1. Body Composition of Combat Athletes

The body composition results for combat athletes are shown in Table 4. In most studies that measure body composition, male and female athletes had normal body mass index (BMI) values, with low or normal fat percentages and adequate, and in some cases high, lean mass levels, both in reference periods and in pre-competitive periods [37,38,42,44,46,47,48,49,50,52,55,56,57,58]. On the other hand, in three of the studies [39,40,41], the athletes’ BMI was found to be above normal weight values while maintaining normal levels of body fat.

Longitudinal studies, i.e., those that allow for variations in athletes’ body composition to be identified between several points in time [53,54,55,56,57,58], show that acute pre-competitive weight loss was common among combat athletes who had a competition weight (at weigh-in) lower than their usual weight. The reduction in body mass occurred mainly at the expense of lean mass, with no significant changes in fat mass [53,55,56,57]. On the other hand, one of the studies [56] reveals that, although in both sexes weight loss occurred mainly at the expense of lean mass, these losses of lean mass were greater in men.

In terms of sex, studies in which the sample consisted of athletes of both sexes [44,46,47,48,49,56] reveal differences between men and women in body characteristics, with men having greater average height and body weight, although with similar body mass indices, as well as greater lean mass and lower body fat percentage than women.

With regard to sporting discipline, jiu-jitsu athletes have the highest average BMI values, with no significant differences in terms of fat mass and lean mass percentages, which are similar to those of other athletes [38,39,41]. On the other hand, in terms of competition level, no differences in body composition were detected among combat sports athletes.

#### 3.2.2. Dietary Intake and Eating Habits of Combat Athletes

The dietary intake results for combat athletes are shown in Table 5. Table 6 presents the degree of compliance with official intake recommendations [8,59] for the different combat athlete samples from the studies included in this review.

After comparing the studies that reported intake values with the official recommendations, only two studies met the energy intake recommendation [39,48] and only one the carbohydrate intake recommendation [39]. In contrast, the rest reported lower values [36,38,42,43,44,45,46,47,48,49,51,53,54,55,57,58]. Low levels of energy and carbohydrate intake were observed both in reference periods, i.e., not necessarily close to a competition, and in pre-competitive periods. Regarding protein, most studies report adequate consumption [36,38,42,43,45,46,47,48,51,53,55,57], although two report an intake above the recommended level [39,44] and only one below [49]. On the contrary, there is considerable controversy regarding fat consumption: in some cases recommendations are met [36,39,43,45,47,57], in others high consumption is observed [46,48,51,53,55,57], and in two studies consumption is below the recommended level [42,44].

With regard to micronutrients, several studies refer to deficiencies in certain vitamins and minerals among combat athletes, especially in pre-competition periods [38,43,44,45,46,47,53,55,57]. However, two studies [44,45] report an intake above the recommendations for some micronutrients.

Five of the studies included in the review [37,41,43,46,47] also analysed the frequency of food consumption among athletes. All five observed fruit and/or vegetable consumption below the recommendations, with two cases [37,46] showing excessive meat consumption as well.

Longitudinal studies, which allow for variation in athletes’ nutritional intake to be identified across several points in time [53,54,55,56,57], report reduced energy and nutrient consumption in the pre-competitive period compared to usual intake.

In terms of sex, sport discipline, and level of competition, no differences in nutritional intake (energy and macronutrients) or eating habits were observed among combat athletes.

### 3.3. Risk of Bias

The methodological quality of the included studies was assessed using the STROBE checklist (Table 7) for observational studies, [33] the Newcastle–Ottawa scale (NOS) for cohort studies [34], and the adapted NOS-xs scale for analytical cross-sectional studies and NOS-xs2 for analytical cross-sectional studies [35] (Table 8 and Supplementary Tables S3–S5). The NOS and adapted scales assessment revealed heterogeneous distribution of methodological quality among the included studies. Of the 23 studies, nine [38,40,41,42,43,46,55,56,57] were classified as having a low risk of bias, indicating greater methodological rigour; 13 studies [36,37,39,44,45,47,48,49,50,52,53,54,58] were categorised as having a moderate risk of bias, suggesting some limitations in their study design; and one study was categorised as having a high risk of bias, suggesting strong limitations in its methodology [51].

## 4. Discussion

The objective of this study was to review the body composition, dietary intake, and eating habits of male and female combat athletes, with the aim of analysing whether they have a body composition that is adequate for proper performance in sport and whether they comply with official dietary recommendations.

This systematic review compiled research on various combat sports, including judo, jiu-jitsu, karate, taekwondo, boxing, kickboxing, and MMA; covering adults or adolescents, men or women; and body composition and dietary intake of amateur, semi-professional, or professional athletes. After analysing the results of the 23 studies included in the review, a trend was observed in body composition and dietary intake among combat athletes. In most studies, athletes had a normal BMI, low or normal fat percentages, and adequate muscle mass. However, despite maintaining proper body composition, most athletes reported energy and carbohydrate intakes below official recommendations. To construct this comprehensive perspective, our methodology incorporated studies that assessed body composition exclusively, alongside those focused solely on dietary intake. Integrating independent evidence from both types of studies was crucial to robustly demonstrate this central paradox: combat athletes are successfully achieving the required physical standards of their sport, yet they are doing so despite following suboptimal and potentially harmful nutritional patterns.

The longitudinal studies included in the review [53,54,55,56,57,58] revealed variation in athlete’s body composition and dietary intake of athletes at different time points. The energy and nutrient intake of athletes was reduced in the pre-competition periods due to rapid weight loss, which occurred mainly at the expense of lean mass without significant changes in fat mass. The reduction in lean mass is mainly due to acute dehydration strategies, which can significantly disrupt electrolyte levels and increase the risk of acute cardiovascular complications, potentially affecting athletes’ health and performance [13].

### 4.1. Body Composition

Body composition assessment plays a key role in monitoring athletes’ performance and training strategies, especially in weight-class sports, where body composition significantly affects performance [10]. In combat sports, where competition is organised by weight class, it is important to maintain low levels of fat mass, as this makes it easier to compete in lower weight classes and increases the chances of success in competitions [60]. In addition, low body fat is associated with better results in various physical performance variables in combat athletes [61,62], as is adequate lean mass [62]. Therefore, taking the above into account, most of the studies included in this review [37,38,42,44,46,47,48,49,50,52,53,55,56,57,58] reveal that combat athletes have adequate body composition for proper athletic performance, regardless of sex, competition level, and sport discipline.

Regarding sex differences, male athletes had a higher BMI and greater lean mass, as well as lower body fat percentage than female athletes, a pattern that has already been reported in elite combat sport populations [63]. This pattern is not unique to combat sports but rather reflects the sexual dimorphism in body composition which has been extensively described in the general population, whereby women show a higher percentage of body fat and men higher fat-free mass [64].

Despite maintaining an adequate body composition, rapid pre-competitive weight loss was common among combat athletes [46,53,54,55,56,57,58]. Leaving aside the controversy regarding the consequences of rapid weight loss on athletic performance, the negative impact of these procedures on athletes’ health is well described in the scientific literature [65]. Therefore, there is a need to change the approach to nutritional interventions for combat athletes leading up to a competition. It is recommended to follow nutritional strategies that promote gradual weight loss from fat mass, and to avoid rapid weight loss strategies, which can lead to a multitude of negative consequences [22].

Regarding body mass index (BMI), although most athletes in this review presented values within the “normal” range, it is important to emphasise that BMI is not the most relevant index to consider in athletic populations. This is particularly critical in combat sports characterised by high musculoskeletal development, as BMI does not distinguish between different body compartments (fat, muscle, lean, or bone mass) [66]. Therefore, in these populations, a high BMI may reflect increased muscle mass rather than excess adiposity, which justifies the use of more precise methods like DXA, BIA, or skinfolds included in this review to accurately assess the athletes’ physical status [10,67].

### 4.2. Dietary Intake

The main finding regarding the dietary intake of combat athletes is that their energy and carbohydrate consumption falls below official recommendations [36,38,42,43,44,45,46,47,48,49,51,53,54,55,57,58]. This trend has been observed previously in other types of intermittent sports with similar physiological demands [68]. Low energy availability (LEA) is defined as an imbalance between dietary energy intake and energy expenditure during exercise, leading to a deterioration in the physiological function of multiple organ systems [69]. LEA affects both men and women, being particularly prevalent in the latter, and is very common among combat athletes in weight categories. Cyclical changes in body mass and composition, among other factors, are one of the main contributors to LEA [70]. The effects of LEA have been primarily described in female athletes, in whom it has a greater impact, and only recently in male athletes [70]. LEA negatively affects recovery, muscle mass, and neuromuscular function and increases the risk of injuries and illnesses that can compromise athletic performance [71].

The LEA has mainly been caused by suboptimal carbohydrate intake. Depletion of glycogen stores through reduced carbohydrate intake is a common tactic in rapid weight loss strategies [17]. These strategies of reducing carbohydrate intake in combat sports, which are intermittent sports involving high-intensity efforts, can lead to performance declines [72].

On the other hand, regarding protein intake, most studies report adequate consumption [36,38,42,43,45,46,47,48,51,53,55,57,58]. Adequate protein intake contributes to the maintenance of muscle mass [73] and prevents or reduces catabolic consequences of inadequate energy intake [74]. Likewise, the co-intake of adequate amounts of protein and carbohydrates in the post-exercise period may be beneficial for glycogen resynthesis [75], given that combat sports, which include aerobic and anaerobic metabolism, can deplete muscle glycogen stores during exercise [6].

With regard to fat consumption, there has been considerable controversy and diversity among combat athletes. Although carbohydrates are the main source of energy for combat sports athletes [6], fats also play many important roles. Very low fat diets can lower testosterone levels in men, but more studies are needed in this area [76]. Conversely, high fat consumption can compromise an athlete’s performance, especially during pre-competitive periods, as it can cause gastrointestinal discomfort [77].

Regarding sex, no significant differences in nutritional intake (energy and macronutrients) were observed among combat athletes, suggesting that inadequate energy and carbohydrate intake is common among both male and female athletes [78]. Basic combat sport nutrition (energy, macronutrients, and weight cutting) is similar for men and women, but women generally need stricter protection of energy availability, iron, calcium, vitamin D, and cycle-aware planning and may require more conservative weight-cut strategies and closer monitoring [79]. Despite these differences, most nutritional recommendations in combat sports are not sex-specific [8,80].

In terms of food, this review reveals a deficient consumption of fruit and/or vegetables by combat athletes [37,41,43,46,47]. Daily intake of fruit and vegetables can prevent major non-communicable diseases, such as cancer and cardiovascular disease, as well as providing the body with dietary fibre and a wide variety of micronutrients [81]. Studies that use only a single 24 h dietary recall or a one-day dietary record to assess dietary intake are not representative of habitual intake, and no conclusions can be drawn about their impact on health or performance.

Finally, the methods used to assess dietary intake in the included studies are validated, but they have some limitations that may lead to misreporting of dietary intake. Prospective methods (3–7-day diet records) may alter habitual intake patterns, whereas retrospective methods (24 h recalls or food frequency questionnaires) rely on athletes’ memory and may introduce recall bias in the type and quantity of foods consumed [82]. Athletes may change their eating habits over time, whether due to changes in their body composition goals, nutritional needs, or professional recommendations, among other reasons. The different methods may not capture these changes or require periodic updates to reflect changing consumption.

### 4.3. Limitations

The studies included in this systematic review involved some with small sample sizes, and their results may be difficult to extrapolate to other populations. The instruments used to measure body composition were diverse: some employed anthropometry, others BIA, and others DXA, so the accuracy of measurements may vary depending on sensitivity of these instruments. Additionally, each method relies on different assumptions and prediction models; consequently, the resulting body composition values are not directly comparable across studies, and any between-study differences should be interpreted cautiously [67]. Furthermore, not all studies that used the anthropometric method provide details about the measurement protocol they applied or the number of measurements performed, so the methodology and anthropometrists could be biased. Studies that used the ISAK protocol cite the first version of this protocol, which has been updated over time [22]. Similarly, the instruments used to measure dietary intake were dietary surveys (24 h recalls or 3- or 7-day records). These instruments have a large margin of error when estimating intake. In addition, to ensure that intake data are representative of habitual intake, at least three 24 h dietary recalls or a three-day dietary record (or longer) should be used. Another key limitation is the variability in assessment timing. In combat sports, proximity to competition can induce acute changes in body composition and dietary intake estimates; therefore, results from non-equivalent periods are not directly comparable.

Further research is needed to determine whether the current recommendations are adequate for combat sports, as athletes do not comply with them yet still have adequate body composition compatible with proper athletic performance. It would also be necessary to establish sex-specific nutritional recommendations.

## 5. Conclusions

This systematic review found that, despite maintaining adequate body composition, combat athletes reported an inadequate dietary pattern in accordance with official recommendations, especially in pre-competitive periods, which can negatively influence athletic performance. Considering the negative impact that the loss of lean mass associated with rapid weight loss can have on athletes’ health and performance, the authors highlight the importance of following safe nutritional strategies that do not lead to nutritional deficiencies and do not pose a risk to athletes’ health. It should also be noted that dietary intake recommendations for athletes are not differentiated by sex, highlighting the need for further research to establish specific dietary intake recommendations for each sex and type of combat sport.

## Figures and Tables

**Table 1:** The inclusion criteria applied in the study followed the Population, Intervention, Comparison, and Outcomes (PICO) strategy.

Population	Intervention	Comparation	Outcomes
Amateur, semi-professional, or professional athletes in boxing, karate, kickboxing, jiu-jitsu, taekwondo, judo, muay thai, and MMA. Sex: male or female. Healthy athletes. Adults and adolescents (aged 12–65 years).	Body composition analysis. Dietary intake surveys or nutritional interviews.	Sex. Level of competition. Sports. Compliance with nutritional requirements.	Body composition data: BMI, percentages, and values of fat mass and lean mass. Dietary intake data: energy, carbohydrates, proteins, and fats.

MMA: mixed martial arts.

**Table 2:** Keywords and MeSH terms used in search strategy.

Concept	Keywords and/or MeSH Terms
Combat sports	“Martial arts” OR “Boxing” OR “karate” OR “kickboxing” OR “jiu jitsu” OR “brazilian jiu jitsu” OR “taekwondo” OR “judo” OR “muay thai”
Dietary intake	“Feeding behavior” OR “dietary habit” OR “food consumption” OR “Diet”
Body composition	“Anthropometry” OR “anthropometric characteristics” OR “anthropometric variables” OR “Body composition” OR “Skinfold Thickness”
PubMed search strategy *	
(“Martial arts”[MeSH Terms] OR “martial arts”[Title/Abstract] OR “Boxing”[MeSH Terms] OR “boxing”[Title/Abstract] OR “karate”[Title/Abstract] OR “kickboxing”[Title/Abstract] OR “jiu jitsu”[Title/Abstract] OR “brazilian jiu jitsu”[Title/Abstract] OR “taekwondo”[Title/Abstract] OR “judo”[Title/Abstract] OR “muay thai”[Title/Abstract]) AND (“Feeding behavior”[MeSH Terms] OR “feeding behavior”[Title/Abstract] OR “dietary habit”[Title/Abstract] OR “food consumption”[Title/Abstract] OR “Diet”[MeSH Terms]) AND (“Anthropometry”[MeSH Terms] OR “anthropometry”[Title/Abstract] OR “anthropometric characteristics”[Title/Abstract] OR “anthropometric variables”[Title/Abstract] OR “Body composition”[MeSH Terms] OR “body composition”[Title/Abstract] OR “Skinfold Thickness”[MeSH Terms] OR “skinfold”[Title/Abstract])	

* The search strategy was adapted for each of the databases consulted using the Polyglot Search tool from the Systematic Review Accelerator [30] (Appendix A).

**Table 3:** Characteristics of the included studies.

Author and Year	Study Design	Objective	Sample	Analysis/Intervention	Main Outcomes	Conclusions
Filaire et al., 2001 [53]	Cohort	Assess the physiological and dietary profile of judo athletes during a baseline period and a weight loss period.	11 male judokas from the Auvergne league.	Body composition (anthropometry) and dietary intake (7-day record).	Normal BMI, normal body fat levels, and high lean mass during the baseline period. There was a reduction in body mass during the weight loss period at the expense of lean mass. Energy, carbohydrate, and micronutrient intake was reduced during the weight loss period.	The athletes had an adequate body composition in the reference period, decreasing their body mass at the expense of lean mass in the weight loss period. Carbohydrate and micronutrient intake was below recommendations in both periods.
Teshima et al., 2002 [47]	Cross-sectional	Compare the nutrient intake of male and female university karate athletes.	45 highly competitive Japanese university karate athletes (Group M: 29 men and Group F: 16 women).	Body composition (anthropometry) and dietary intake (3-day record).	Men had greater height, average body weight, and lean mass than women, with a lower percentage of fat. Both men and women had energy intakes below recommendations, as well as other nutrients. Consumption of vegetables, milk, and dairy products was low in both groups.	The highly competitive male and female karatekas studied in this study may be at risk of suboptimal nutrient intake, increasing the potential for nutrient deficiencies.
Fleming & Costarelli, 2007 [55]	Cohort	Investigate the nutritional intake and body composition of taekwondo athletes during a two-week pre-competition period.	Seven male taekwondo athletes, aged 17 to 28.	Body composition (anthropometry) and dietary intake (3-day record in reference period and 5-day record in pre-competition period).	Reduction in body weight, without significant reduction in fat mass and with reduction in lean mass. Energy, carbohydrate, calcium, zinc, and water intake below recommendations, both normally and before competition, and fat and salt consumption exceeded recommendations.	Taekwondo athletes had a low percentage of fat and weight loss at the expense of lean mass. Athletes consumed a suboptimal diet both normally and in the pre-competition period.
Prouteau et al., 2007 [56]	Cohort	To provide an accurate quantification of soft tissue alterations and analyse sex differences in response to weight cuts in elite judokas.	48 elite judokas (men *n* = 22; women *n* = 26) from the French national training camp in Orleans.	Body composition (DXA). Dietary intake data have not been reported.	Normal BMI in men and women, with normal fat percentage, at the start of the season. Athletes who practised rapid weight loss and gain suffered alterations in body composition in the pre- and post-competitive periods.	Male and female judokas had adequate body composition at the start of the season, with variations in the pre- and post-competitive periods in the case of athletes who modified their weight to compete.
Ubeda et al., 2010 [37]	Cross-sectional	Assess the dietary intake, eating habits, and body composition of elite combat athletes.	22 male athletes from the Spanish National Taekwondo, Judo and Boxing Teams.	Body composition (DXA) and food consumption and eating habits questionnaire.	Average BMI = 22.7 kg/m^2^. Body fat percentage = 9.25%. Intake of vegetables, cereals, bread, rice, potatoes, and pasta was lower than recommended, while intake of red meat and meat products exceeded recommendations.	Combat athletes had adequate body composition, excessive intake of food groups that provide more protein and lipids, and low intake of the main dietary sources of carbohydrates and fibre.
Zonta et al., 2011 [42]	Cross-sectional	Assessing the dietary and anthropometric profile of male judo competitors.	Eleven male judo athletes, aged between 15 and 32, from a team in western Santa Catarina.	Body composition (anthropometry) and dietary intake (24 h recall).	Average BMI = 24.8 kg/m^2^. Body fat = 10.9%. Average energy intake (2399.29 kcal), carbohydrate intake (58.36% VCT), and fat intake (20.51% VCT) were below the recommended levels, while protein consumption (1.74 g/kg weight) was within normal limits.	The judokas had an anthropometric profile suitable for the sport. In terms of dietary profile, the judokas had a deficient energy and carbohydrate intake compared to the recommendations.
Catikkas et al., 2013 [50]	Cross-sectional	To assess the kinanthropometric attributes of different combat sports like karate, taekwondo, judo, and kickboxing.	48 national level male athletes from four different sports (karate *n* = 7; taekwondo *n* = 15; judo *n* = 11; and kickboxing *n* = 15).	Body composition (anthropometry). Dietary intake data have not been reported.	Young male combat sport athletes showed normal weight profiles with low body fat. Their build was predominantly mesomorphic, with broad shoulders, narrow hips, and a medium-sized trunk.	Young combat sports athletes have normal BMI values and low body fat percentage. These athletes are meso-ectomorphs.
Książek et al., 2014 [51]	Cross-sectional	To assess the energy value and the intakes of nutrients, minerals, vitamins, dietary fibre, and water in daily food rations including and excluding supplements among male judo athletes.	28 professional male judo athletes (13 classified as first sport class, 12 as champions class, and 3 as international champions class).	Dietary intake (5-day recall method). Body composition data have not been reported.	Energy intake was below recommended levels, and carbohydrate intake was insufficient for the athletes’ training demands.	Overall, the athletes’ diets appeared energy- and carbohydrate-deficient relative to training demands, while protein intake was already sufficient.
Pettersson & Berg, 2014 [36]	Cross-sectional	Investigate the dietary intake of combat athletes in Olympic disciplines between weigh-in and the first bout.	68 elite Swedish athletes (21 women and 47 men) from the disciplines of wrestling, taekwondo, judo, and boxing.	Dietary intake (1-day record). Body composition data have not been reported.	Carbohydrate, protein, and fat intake levels were 5.5 ± 3.5, 1.4 ± 0.8, and 1.1 ± 0.8 g/kg body weight, respectively. Average water intake was 55 ± 33 mL/kg body weight. There were no significant differences in nutritional intake between the sexes.	The average carbohydrate intake among combat athletes was below current recommendations for sports nutrition.
Reljic et al., 2015 [57]	Cohort	To evaluate dietary intake, vitamin status, and oxidative stress in elite male boxers.	17 elite male boxers (RWL group: 10 participants and control group: 7 participants).	Body composition (anthropometry) and dietary intake (7-day record) during a reference week, one week prior to competition, and one week after competition.	RWL group: significant changes in body weight and lean mass between the three time points. Energy and nutrient intake was reduced during the week prior to competition in the RWL group. Both groups showed a negative energy balance in all three periods, with intakes of carbohydrates, vitamin A, vitamin E, and folate below the recommended values.	Elite boxers had a low-calorie, low-carbohydrate diet, regardless of whether or not they participated in rapid pre-competition weight loss.
Andreato et al., 2016 [39]	Cross-sectional	Analyse the physiological, nutritional, and performance profiles of athletes practising Brazilian jiu-jitsu.	15 adult male Brazilian jiu-jitsu athletes (eight brown belts and seven black belts).	Body composition (anthropometry) and dietary intake (24 h recall)	Average BMI = 25.60 kg/m^2^. % Body fat = 12.7%. High muscle development (59.2%) and somatotype dominated by mesomorphic component. Average intake of 46.4 kcal/kg, 6.3 g CH/kg, 2.2 g protein/kg, and 1.4 g lipids/kg.	Jiu-jitsu athletes had low-to-average levels of body fat, high muscle mass, and a mesomorphic somatotype. The athletes had a high protein intake.
Matthews & Nicholas, 2017 [54]	Cohort	Quantify the magnitude and identify methods of rapid weight loss and rapid weight gain in MMA athletes preparing for competition.	7 male MMA athletes.	Dietary intake (7-day record per weigh-in). Body composition data have not been reported.	Body mass decreased during the week prior to weigh-in and increased after weigh-in. Energy and carbohydrate intake was low during the week prior to weigh-in, increasing from weigh-in to competition.	MMA athletes practised rapid weight loss prior to competition through low energy and carbohydrate intake and dehydration (among other methods).
Książek et al., 2017 [45]	Cross-sectional	To evaluate the energy and nutrient intake of representatives of the Polish national judo team during the pre-competition period.	30 elite judokas (15 women and 15 men).	Dietary intake (7-day record). Body composition data have not been reported.	Average daily intake of energy, carbohydrates, fats, fibre, water, potassium, calcium, iron, iodine, and vitamins D, C, E, and B2 was lower than the recommended standard. On the other hand, dietary intake of sodium, phosphorus, and vitamins B3, B6, and B12 was significantly higher than recommended.	Judo athletes showed reduced intake of energy, carbohydrates, and nutrient diversity in the pre-competitive period.
Papadopoulou et al., 2017 [46]	Cross-sectional	To investigate and evaluate common diet and weight control strategies among taekwondo athletes prior to national competitions.	60 taekwondo athletes (23 women and 37 men).	Body composition (BIA) and dietary intake (3-day record).	Significant differences between men and women in body characteristics (both normal BMI and low body fat percentage). Neither men nor women meet their energy and/or intake requirements according to recommendations.	Taekwondo athletes were severely malnourished before the competition, with a significant negative energy balance and intakes below the recommended levels for most macro- and micronutrients.
Ribas et al., 2017 [44]	Cross-sectional	To determine the dietary profile of Brazilian national team karatekas during the pre-competitive period.	19 karatekas (10 men and 9 women), aged between 18 and 30 years.	Body composition (anthropometry) and dietary intake (3-day record).	Both male and female karatekas had low levels of body fat and high lean mass. In terms of dietary profile, both men and women had a high-protein, low-glycaemic, and low-lipid profile.	The karate athletes in the sample had an inadequate diet in terms of macronutrients.
Anyzewska et al., 2018 [43]	Cross-sectional	To assess the prevalence of rapid weight loss and identify potential dietary deficiencies among professional martial arts athletes.	62 male combat sports athletes (judo, kickboxing, jiu-jitsu, MMA, and boxing) from professional sports clubs in Poland.	Frequency of food consumption and dietary intake (24 h recall). Body composition data have not been reported.	Consumption levels of dairy products, cereals, fruit, and vegetables below recommendations. Insufficient intake of energy (2377 kcal), carbohydrates (3.6 g/kg body weight), minerals (especially iodine, potassium, and calcium), and vitamins (especially D, folate, C, and E).	Combat athletes had insufficient intake of energy, carbohydrates, and some vitamins and minerals, with adequate intake of protein and fat. In addition, they did not meet the intake recommendations for some food groups.
Villarroel et al., 2018 [41]	Cross-sectional	Assess the anthropometry and analyse the frequency of food consumption of male jiu-jitsu athletes.	25 male jiu-jitsu athletes, aged between 23 and 47, from an academy in San Bernardo do Campo, Sao Paulo.	Body composition (anthropometry) and frequency of food consumption.	Average BMI = 27.63 kg/m^2^. Body fat = 17.40%. Food consumption frequency: 84% reported consuming carbohydrates and protein sources daily, and 56% reported consuming fat sources daily. Sixty percent reported not consuming fruit daily, and 64% reported not eating vegetables daily.	The jiu-jitsu athletes had an overweight BMI and a normal body fat percentage, but they had a deficient intake of fruit and vegetables.
da Luz & da Rosa, 2019 [38]	Cross-sectional	Assess the nutritional profile of jiu-jitsu fighters.	23 male jiu-jitsu athletes with an average age of 25 years.	Body composition (anthropometry) and dietary intake (3-day record).	Average BMI = 24.80 kg/m^2^. Body fat = 8.44%. Average intake of 2271 kcal/day, 3.13 g CH/kg, 1.62 g protein/kg, and 1.13 g lipids/kg. Intake of vitamin C, calcium, and magnesium below recommendations.	The jiu-jitsu athletes had a body composition suitable for proper performance in the sport; however, they showed a deficient intake of energy, carbohydrates, and some micronutrients.
Dimitrijevic et al., 2022 [52]	Cross-sectional	To explore the correlations of different developed anthropometric equations with DXA measurements as well as the correlations of BIA with DXA measurements in determining the percentage of body fat in male athletes.	101 male athletes recruited from three different combat sports: wrestling (*n* = 33), judo (*n* = 35), and kickboxing (*n* = 33).	Body composition (DXA). Dietary intake data have not been reported.	DXA indicated generally lean physiques, with kickboxers showing slightly higher mean body fat than the other groups.	The authors conclude that selected anthropometric equations are practical and affordable alternatives for routine % body fat assessment when DXA/BIA are not feasible in combat sports contexts.
Niewczas et al., 2023 [40]	Cross-sectional	Analyse the overall and segmental body components of kickboxing athletes immediately before a sporting event.	30 high-level kickboxing athletes.	Body composition (BIA). Dietary intake data have not been reported.	Normal levels of indicators describing body composition, except for a slight exceedance of normal values for skeletal muscle mass and BMI (25.19 kg/m^2^). High symmetry between limbs and adequate body water content.	The kickboxing athletes presented a body composition in accordance with the standards for a healthy person. The athletes had adequate body weight, good body hydration, and adequate mineral and protein content.
Baranauskas et al., 2024 [48]	Cross-sectional	To determine the somatotype profiles in association with body composition and nutritional profiles among Lithuanian elite athletes involved in water, cycling, and combat sports.	59 combat sports athletes (52 men and 7 women). Boxing *n* = 14; Taekwondo *n* = 4; Wrestling *n* = 29; Judo *n* = 12.	Body composition (BIA) and dietary intake (3-day record).	Athletes exhibited a lean, muscular phenotype, with women showing higher adiposity than men. Dietarily, their pattern suggested insufficient carbohydrate intake, alongside a relatively high fat contribution.	Combat sports athletes predominantly show a mesomorphic physique with an endomorphic tendency, consistent with sport-specific adaptation. Somatotype aligns with habitual dietary patterns
Liang et al., 2025 [58]	Cohort	To examine the prevalence of LEA in Chinese female combat athletes and monitor changes in physiological function and performance during the pre-competition period.	11 female judo athletes.	Body composition (DXA) and dietary intake (3-day record per weigh-in).	The week before competition, body weight and body fat percentage were reduced compared with earlier weeks. Dietary intake showed a drop in carbohydrate intake, while the overall macronutrient distribution remained broadly similar between groups.	During the weight-loss phase, athletes generally reduced body weight and body fat while largely maintaining muscle mass, but the accompanying LEA was associated with unfavourable physiological changes as competition approached.
Samanipour et al., 2025 [49]	Cross-sectional	To examine the differences in body composition, psychological skills, nutrient intake, physical performance, and their correlations in a sample of young male and female taekwondo athletes.	35 Iranian National team taekwondo athletes (20 males and 15 females).	Body composition (BIA) and dietary intake (24 h dietary recalls and 7-day open food report).	In adolescent taekwondo athletes, boys exhibited higher fat-free mass and greater skeletal muscle mass, along with higher fat mass. In dietary intake, boys reported a higher relative energy intake and greater fat consumption, while protein and carbohydrate intakes were similar.	In boys, greater muscle mass and higher energy intake were associated with better maintenance of performance in the final rounds. In girls, lower fat intake and different nutrient–performance relationships were observed.

DXA = dual-energy X-ray absorptiometry; F = female; BMI = body mass index; LEA = low energy availability; M = male; MMA = mixed martial arts; RWL = rapid weight loss.

**Table 4:** Outcomes of body composition of combat athletes.

Author and Year	Sample Size (*n*)	Age (Years)	Sport Modality	Measurement Instruments	Protocol	Season Phase	Body Mass (kg)	Stretch Stature (cm)	BMI (kg/m^2^)	Fat Mass (%)	Fat Mass (kg)	Fat-Free Mass (kg)	Muscle Mass (kg)
Filaire et al., 2001 [53]	T = 11 (M: 11; F: 0)	-	Judo	PLI Harpenden	-	Reference period	75.1 ± 2.6	172.9 ± 1.5	24.9 ± 1.2	17.3 ± 2.1 ^DR^	-	62.1 ± 2.2	-
						Pre-competition (−7)	71.5 ± 1.3	172.9 ± 1.5	23.1 ± 0.7	16.8 ± 1.4 ^DR^	-	59.8 ± 2.7	-
Teshima et al., 2002 [47]	T = 45 (M: 29; F: 16)	M = 20.1 ± 1.3 F = 19.7 ± 1.0	Karate	PLI Harpenden	3 measurements	Reference period (M)	66.2 ± 7.5	173.6 ± 6.4	22.0 ± 2.0	12.4 ± 3.0 ^DR,S^	8.3 ± 2.8	57.9 ± 5.5	-
						Reference period (F)	56.3 ± 6.8	158.9 ± 4.9	22.3 ± 2.6	24.1 ± 5.5 ^DR,S^	13.7 ± 4.3	42.6 ± 4.5	-
Fleming & Costarelli, 2007 [55]	T = 7 (M: 7; F: 0)	20.1 ± 4.6	TKD	BAS Seca, PLI Holtain	BASES (1997) and Norton et al. (2002) *	Reference period	65.2 ± 11.5	172.0 ± 7.7	21.9 ± 2.4	3.2 ± 0.8 ^JP^	2.3 ± 0.9	62.4 ± 11.2	-
						Pre-competition (−5)	64.4 ± 11.6	172.0 ± 7.7	21.6 ± 2.4	3.2 ± 0.8 ^JP^	2.2 ± 1.0	61.9 ± 11.4	-
Prouteau et al., 2007 [56]	T = 48 (M: 22; F: 26)	M = 21.0 ± 3.0 F = 19.0 ± 2.0	Judo	DXA Hologic QDR 4500 Enhanced Array Whole Body software 11.2	-	Reference period (M)	73.5 ± 8.0	174.9 ± 4.9	24.0 ± 2.0	11.6 ± 3.6	-	61.6 ± 5.8	-
						Reference period (F)	60.2 ± 9.0	162.8 ± 7.3	22.6 ± 2.4	23.0 ± 4.0	-	43.7 ± 5.0	-
Ubeda et al., 2010 [37]	T = 22 (M: 22; F: 0); TKD = 8; JUD = 8; BOX = 6	TKD = 17–32; JUD = 16–26; BOX = 19–30	TKD, Judo, and Boxing	BAS Seca, EST Seca, DXA Norland XR-46	-	Reference period	72.1 ± 14.7	180.0 ± 5.0	22.8 ± 4.9	9.0 ± 7.0	-	-	-
Zonta et al., 2011 [42]	T = 11 (M: 11; F: 0)	22.0 ± 5.3	Judo	BAS Techline, EST Sanny, PLI Sanny	3 measurements	Reference period	74.7 ± 12.9	174.0 ± 6.0	24.8 ± 3.2	10.9 ± 4.0 ^JP^	-	-	-
Catikkas et al., 2013 [50]	T = 48 (M: 48; F: 0); Karate = 7; TKD = 15; JUD = 11; KBX = 15	20.3 ± 3.19	Karate, TKD, Judo, and KBX	BAS Angel, 150 MA PLI Holtain	-	Reference period	67.4 ± 10.6	174.5 ± 7.2	22.0 ± 2.7	12.2 ± 3.1 ^Y^	-	-	-
Reljic et al., 2015 [57]	T = 17 (M: 17; F: 0); CG = 7; RWLG = 10	CG = 18.4 ± 2.2RWLG = 19.7 ± 3.2	Boxing	BAS Seca, EST Seca, PLI Holtain	Lohman et al. (1998) *	Reference period (CG)	64.8 ± 8.9	174.6 ± 7.4	21.3 ± 3.4	9.5 ± 2.1 ^L^	-	58.5 ± 7.1	-
						Reference period (RWLG)	67.4 ± 9.4	175.5 ± 7.0	21.9 ± 3.5	8.7 ± 1.7 ^L^	-	61.4 ± 8.1	-
						Pre-competition (CG) (−7)	65.0 ± 9.5	174.6 ± 7.4	21.3 ± 3.6	9.5 ± 2.0 ^L^	-	58.7 ± 7.5	-
						Pre-competition (RWLG) (−7)	63.7 ± 9.3	175.5 ± 7.0	20.7 ± 3.4	8.2 ± 1.6 ^L^	-	58.4 ± 8.1	-
Andreato et al., 2016 [39]	T = 15 (M: 15; F: 0)	28.0 ± 5.0	Jiu-jitsu	PLI Harpenden	Lohman et al. (1998) *	Reference period	80.3 ± 7.8	177.5 ± 6.4	25.6 ± 2.9	12.7 ± 4.8 ^S,JP^	10.5 ± 5.2	69.8 ± 4.3	47.5 ± 5.8
Papadopoulou et al., 2017 [46]	T = 60 (M: 37; F: 23)	M = 20.4 ± 3.6 F = 19.4 ± 2.9	TKD	EST Seca 220, BAS Seca 707, BIA Bodystat 1500	-	Pre-competition (M) (−3)	68.3 ± 9.8	170.0 ± 10.0	21.1 ± 1.9	8.5 ± 2.8	5.9 ± 2.5	62.4 ± 8.4	-
						Pre-competition (F) (−3)	52.7 ± 5.7	160.0 ± 10.0	19.4 ± 2.2	13.4 ± 4.2	7.1 ± 2.7	45.4 ± 4.5	-
Ribas et al., 2017 [44]	T = 19 (M: 10; F: 9)	M = 24.5 ± 2.4 F = 25.4 ± 2.7	Karate	BAS Filizola, EST Seca, PLI Lange	Lohman, Roche and Martorell (1988) *	Pre-competition (M)	77.5 ± 11.2	177.2 ± 8.6	24.7 ± 4.3	7.0 ± 2.1 ^JP^	5.6 ± 2.4	71.0 ± 9.2	-
						Pre-competition (F)	61.5 ± 7.1	163.7 ± 3.8	22.9 ± 2.9	15.2 ± 2.5 ^JP^	9.1 ± 2.5	51.6 ± 4.9	-
Villarroel et al., 2018 [41]	T = 25 (M: 25; F: 0)	23–47	Jiu-jitsu	BAS Welmy, EST Sanny	-	Reference period	89.4 ± 18.3	181.0 ± 7.0	27.6 ± 4.5	17.4 ± 5.7 ^JP^	-	-	-
da Luz & da Rosa, 2019 [38]	T = 23 (M: 23; F: 0)	25.0 ± 2.9	Jiu-jitsu	PLI Prime Med A10, BAS Work Hard Dream Big, EST Corporal Wiso	3 measurements	Reference period	-	-	24.8 ± 2.2	8.4 ± 2.8 ^JP^	-	-	-
Dimitrijevic et al., 2022 [52]	T = 101 (M: 101; F: 0); WRS = 33; JUD = 35; KBX= 33	20.9 ± 4.2	Wrestling, Judo, and KBX	DXA (Lunar iDXA scanner)	-	Reference period	80, 0 ± 14.0	179.8 ± 7.8	24.0 ± 3.3	17.0 ± 5.7	-	-	-
Niewczas et al., 2023 [40]	T = 30 (M: 30; F: 0)	24.1 ± 4.3	KBX	BIA InBody 770	-	Reference period	80.5 ± 10.4	179.0 ± 4.9	25.2 ± 3.7	18.4 ± 7.2	15.4 ± 7.8	65.2 ± 5.3	37.0 ± 3.2
Baranauskas et al., 2024 [48]	T = 59 (M: 52; F: 7); BOX = 14; TKD = 4; WRS = 29; JUD = 12	18.0 ± 3.8	Boxing, TKD, Wrestling, and Judo	BIA X-scan instrument via sending multi-frequency currents of 5, 50, 250, 550, and 1000 kHz	-	Reference period (M)	69.9 ± 17.1	170.0 ± 10.0	-	15.2 ± 5.4	11.4 ± 6.5	58.5 ± 11.7	54.4 ± 10.8
						Reference period (F)	57.2 ± 4.3	160.0 ± 10.0	-	23.2 ± 3.7	13.3 ± 3.0	43.9 ± 2.7	40.7 ± 2.6
Liang et al., 2025 [58]	T = 11 (M: 0; F: 11); LEA = 6; non-LEA = 5	-	Judo	DXA (Lunar iDXA, GE Healthcare, Madison, WI, USA)	-	Pre-competition (LEA) (−7)	60.0 ± 4.8	-	-	20.2 ± 4.7	-	45.24 ± 5.89	-
						Pre-competition (non-LEA) (−7)	55.0 ± 4.6	-	-	19.6 ± 1.7	-	42.50 ± 4.33	-
Samanipour et al., 2025 [49]	T = 35 (M: 20; F: 15)	M = 13 ± 1 F = 13 ± 1	TKD	BIA InBody 720	-	Reference period (M)	56.4 ± 9.5	164.9 ± 11.8	20.6 ± 1.0	6.3 ± 0.4	3.5 ± 0.7	42.8 ± 2.9	31.1 ± 2.2
						Reference period (F)	52.1 ± 8.3	160.5 ± 13.4	20.1 ± 1.1	5.6 ± 0.5	2.9 ± 0.7	36.3 ± 1.6	28.2 ± 1.6

BAS = scale (to assess body mass); BIA = bioelectrical impedance analysis; BMI = body mass index; BOX = boxing; CG = control group; DR = Durnin & Rahaman, 1967; DXA = Dual X-ray Absorptiometry; EST = stadiometer (to assess stretch stature); F = female; JP = Jackson & Pollock, 1978; JUD = judo; KBX = kickboxing; L = Lohman, 1981; LEA = low energy availability; M = male; PLI = skinfold calliper (to assess skinfolds); RWLG = rapid weight loss group; S = Siri, 1961; T = total; TKD = taekwondo; WRS = wrestling; Y = Yuhazs, 1974. * The protocols described by the authors led to the international protocol for anthropometric assessment of the International Society for the Advancement of Kinanthropometry (ISAK).

**Table 5:** Outcomes of dietary intake of combat sports athletes.

Author and Year	Sample Size (*n*)	Age (Years)	Sport Modality	Measurement Instruments	Season Phase	Energy (kcal/kg/Day)	CHO (g/kg/Day)	Protein (g/kg/Day)	Lipids (%)
Filaire et al., 2001 [53]	T = 11 (M: 11; F: 0)	-	Judo	7-day diet record. SCDA Nutrisoft (Bil- nut.4 software package, France).	Reference period	40.3 ± 4.0	4.8 ± 0.6	1.7 ± 0.2	35.5 ± 3.0
					Pre-competition (−7)	29.4 ± 2.6	3.3 ± 0.4	1.2 ± 0.1	37.4 ± 2.4
Teshima et al., 2002 [47]	T = 45 (M: 29; F: 16)	M = 20.1 ± 1.3F = 19.7 ± 1.0	Karate	3-weekday diet record.	Reference period (M)	42.3 ± 12.7	5.7 ± 1.8	1.4 ± 0.5	26.8 ± 5.8
					Reference period (F)	34.9 ± 8.1	4.8 ± 1.1	1.2 ± 0.4	30.0 ± 5.6
Fleming & Costarelli, 2007 [55]	T = 7 (M: 7; F: 0)	20.1 ± 4.6	Taekwondo	3-day food diary. 5-day food diary.	Reference period	34.6 ± 14.5	3.8 ± 1.6	1.2 ± 0.4	40.2 ± 4.4
					Pre-competition (−5)	22.7 ± 8.5	2.5 ± 1.0	1.1 ± 0.5	35.9 ± 5.4
Zonta et al., 2011 [42]	T = 11 (M: 11; F: 0)	22.0 ± 5.3	Judo	24 h record.	Reference period	32.1 ± 13.1	4.7 ± 1.9	1.7 ± 0.7	20.5 ± 5.8
Książek et al., 2014 [51]	T = 28 (M: 28; F: 0)	20.9 ± 3.1	Judo	5-day recall method. Dieta 5.0 software.	Reference period	-	4.5 ± 1.1	1.8 ± 0.5	32.3 ± 5.8
Pettersson & Berg, 2014 [36]	T = 68 (M: 47; F: 21)	M = 21.0 ± 3.1F = 21.9 ± 5.1	Wrestling, Taekwondo, Judo, and Boxing	1-day food register. Software package Dietist XP version 3.2.	Pre-competition (−1)	38.5 ± 22.0	5.5 ± 3.5	1.4 ± 0.8	25.7 ± 23.8
Reljic et al., 2015 [57]	T = 17 (M: 17; F: 0); CG = 7; RWLG = 10	CG = 18.4 ± 2.2RWLG = 19.7 ± 3.2	Boxing	7-day food record. Software DGE PC (version 4.0, 2008, German Nutrition Society, Bonn, Germany).	Reference period (CG)	34.0 ± 6.0	4.1 ± 0.8	1.5 ± 0.3	31.8 ± 9.7
					Reference period (RWLG)	32.0 ± 8.0	3.8 ± 1.1	1.5 ± 0.4	36.6 ± 12.4
					Pre-competition (CG) (−7)	31.0 ± 8.0	3.0 ± 1.2	1.3 ± 0.4	29.0 ± 13.8
					Pre-competition (RWLG) (−7)	18.0 ± 7.0	2.2 ± 0.8	0.8 ± 0.4	30.0 ± 19.1
Andreato et al., 2016 [39]	T = 15 (M: 15; F: 0)	28.0 ± 5.0	Jiu-jitsu	24 h food diary, during two non-consecutive days. Nutrilife 8.1^®^ software.	Reference period	46.4 ± 15.8	6.3 ± 2.3	2.2 ± 1.0	27.0 ± 6.0
Matthews & Nicholas, 2017 [54]	T = 7 (M: 7; F: 0)	24.6 ± 3.5	MMA	Seven-day weighed food record (WFR) using calibrated scales (Salter 305 food scales, Salter: UK).	Pre-competition (−7)	18.0 ± 10.5	1.0 ± 1.2	-	-
Książek et al., 2017 [45]	T = 30 (M: 15; F: 15)	22.2 ± 3.3	Judo	7-day recall method. Software Dieta 5.0.	Pre-competition (−7)	24.0 ± 8.9	3.2 ± 1.2	1.4 ± 0.6	26.6 ± 5.8
Papadopoulou et al., 2017 [46]	T = 60 (M: 37; F: 23)	M = 20.4 ± 3.6F = 19.4 ± 2.9	Taekwondo	3-day food register. Food Processor program (Ver 7.4, ESHA Research, Salem, OR; including traditional national foods).	Pre-competition (M) (−3)	28.7 ± 11.1	3.1 ± 1.2	1.3 ± 0.5	38.3 ± 8.4
					Pre-competition (F) (−3)	34.8 ± 9.6	3.9 ± 1.2	1.5 ± 0.4	37.9 ± 4.1
Ribas et al., 2017 [44]	T = 19 (M: 10; F: 9)	M = 24.5 ± 2.4F = 25.4 ± 2.7	Karate	Food survey of three days.	Pre-competition (M)	37.2 ± 9.0	4.5 ± 2.5	2.1 ± 2.5	19.8 ± 10.3
					Pre-competition (F)	32.7 ± 9.7	4.0 ± 2.0	1.9 ± 0.6	21.6 ± 2.7
Anyzewska et al., 2018 [43]	T = 62 (M: 62; F: 0)	23.0 ± 4.0	Judo, Kickboxing, Jiu-jitsu, MMA, and Boxing	24 h dietary recall.	Reference period	28.6 ± 8.9	3.6 ± 1.1	1.6 ± 0.5	28.0 ± 9.0
da Luz & da Rosa, 2019 [38] *	T = 23 (M: 23; F: 0)	25.0 ± 2.9	Jiu-jitsu	3-day food register. Dietsmart software.	Reference period	2271.0 ± 607.2	3.1 ± 1.2	1.6 ± 0.6	1.1 ± 0.4
Baranauskas et al., 2024 [48]	T = 59 (M: 52; F: 7); BOX = 14; TKD = 4; WRS = 29; JUD = 12	18.0 ± 3.8	Boxing, Taekwondo, Wrestling, and Judo	3-day food record analysis. NutriSurvey software	Reference period	47 ± 15	5.5 ± 2.0	1.6 ± 0.6	39.2 ± 7.1
Liang et al., 2025 [58] **	T = 11 (M: 0; F: 11); LEA = 6; non-LEA = 5	-	Judo	Food weighing method (2 training days and 1 rest day).	Pre-competition (LEA) (−7)	-	2.59 ± 0.62	1.17 ± 0.30	0.82 ± 0.23
					Pre-competition (non-LEA) (−7)	-	2.63 ± 0.79	1.44 ± 0.45	1.03 ± 0.53
Samanipour et al., 2025 [49] **	T = 35 (M: 20; F: 15)	M = 13 ± 1 F = 13 ± 1	Taekwondo	24 h dietary recalls and 7-day open food report.	Reference period (M)	32.4 ± 4.6	4.0 ± 0.8	1.1 ± 0.1	1.4 ± 0.3
					Reference period (F)	29.3 ± 3.1	3.9 ± 0.7	1.1 ± 0.1	1.1 ± 0.1

CG = control group; CHO = carbohydrates; F = female; M = male; RWLG = rapid weight loss group; T = total. * In the Luz et al., 2019 [38] study, energy intake data are in kcal/day and fat intake data are in g/kg/day. ** In the Samanipour et al., 2025 [49] and Liang et al., 2025 [58] studies, fat intake data in in g/kg/day.

**Table 6:** Degree of achievement of dietary intake recommendations for athletes [8,59] from the samples of all studies included in this review.

Author and Year	Season Phase	Energy (kcal/kg/Day)	CHO (g/kg/Day)	Protein (g/kg/Day)	Lipids (%)
		45–50	6–10	1.2–2	25–30
Filaire et al., 2001 [53]	Reference period	No (↓)	No (↓)	Yes	No (↑)
	Pre-competition	No (↓)	No (↓)	Yes	No (↑)
Teshima et al., 2002 [47]	Reference period (M)	No (↓)	No (↓)	Yes	Yes
	Reference period (F)	No (↓)	No (↓)	Yes	Yes
Fleming & Costarelli, 2007 [55]	Reference period	No (↓)	No (↓)	Yes	No (↓)
	Pre-competition	No (↓)	No (↓)	Yes	No (↑)
Zonta et al., 2011 [42]	Reference period	No (↓)	No (↓)	Yes	No (↓)
Książek et al., 2014 [51]	Reference period	-	No (↓)	Yes	No (↑)
Pettersson & Berg, 2014 [36]	Pre-competition	No (↓)	No (↓)	Yes	Yes
Reljic et al., 2015 [57]	Reference period	No (↓)	No (↓)	Yes	No (↑)
	Pre-competition	No (↓)	No (↓)	No (↓)	Yes
Andreato et al., 2016 [39]	Reference period	Yes	Yes	No (↑)	Yes
Matthews & Nicholas, 2017 [54]	Pre-competition	No (↓)	No (↓)	-	-
Książek et al., 2017 [45]	Reference period	No (↓)	No (↓)	Yes	Yes
Papadopoulou et al., 2017 [46]	Reference period (M)	No (↓)	No (↓)	Yes	No (↑)
	Reference period (F)	No (↓)	No (↓)	Yes	No (↑)
Ribas et al., 2017 [44]	Reference period (M)	No (↓)	No (↓)	No (↑)	No (↓)
	Reference period (F)	No (↓)	No (↓)	Yes	No (↓)
Anyzewska et al., 2018 [43]	Reference period	No (↓)	No (↓)	Yes	Yes
da Luz & da Rosa, 2019 [38]	Reference period	-	No (↓)	Yes	-
Baranauskas et al., 2024 [48]	Reference period	Yes	No (↓)	Yes	No (↑)
Liang et al., 2025 [58]	Pre-competition (LEA)	-	No (↓)	No (↓)	-
	Pre-competition (non-LEA)	-	No (↓)	Yes	-
Samanipour et al., 2025 [49]	Reference period (M)	No (↓)	No (↓)	No (↓)	-
	Reference period (F)	No (↓)	No (↓)	No (↓)	-

Symbols ↓ and ↑ indicate in what ways athletes do not comply with official intake recommendations.

**Table 7:** STROBE checklist of the included studies.

Item	Filaire et al., 2001 [53]	Teshima et al., 2002 [47]	Fleming & Costarelli, 2007 [55]	Prouteau et al., 2007 [56]	Ubeda et al., 2010 [37]	Zonta et al., 2011 [42]	Catikkas et al., 2013 [50]	Książek et al., 2014 [51]	Pettersson & Berg, 2014 [36]	Reljic et al., 2015 [57]	Andreato et al., 2016 [39]	Matthews & Nicholas, 2017 [54]	Książek et al., 2017 [45]	Papadopoulou et al., 2017 [46]	Ribas et al., 2017 [44]	Anyżewska et al., 2018 [43]	Villarroel et al., 2018 [41]	da Luz & da Rosa, 2019 [38]	Dimitrijevic et al., 2022 [52]	Niewczas et al., 2023 [40]	Baranauskas et al., 2024 [48]	Liang et al., 2025 [58]	Samanipour et al., 2025 [49]
1.a																		X			X		X
1.b	X	X	X	X	X	X	X	X	X	X	X	X	X	X	X	X	X	X	X	X	X	X	X
2	X	X	X	X	X	X	X	X	X	X	X	X	X	X	X	X	X	X	X	X	X	X	X
3	X	X	X	X	X	X	X	X	X	X	X	X	X	X	X	X	X	X	X	X	X	X	X
4	X		X	X		X				X		X		X	X	X	X	X	X	X	X	X	X
5	X	X	X	X	X	X	X	X	X	X	X	X	X	X	X	X	X	X	X	X	X	X	X
6.a	X	X	X	X	X	X	X	X	X	X	X	X	X	X	X	X	X	X	X	X	X	X	X
6.b		-			-	-	-	-	-		-		-	-	-	-	-	-	-	-	-		-
7	X	X	X	X	X	X	X	X	X	X	X	X	X	X	X	X	X	X	X	X	X	X	X
8	X	X	X	X	X	X	X	X	X	X	X	X	X	X	X	X	X	X	X	X	X	X	X
9	X		X	X	X	X				X	X	X	X	X	X	X			X	X			X
10										X				X		X				X	X		
11	X	X	X	X	X	X	X	X	X	X	X	X	X	X	X	X	X	X	X	X	X	X	X
12.a	X	X	X	X	X	X	X	X	X	X	X	X	X	X	X	X	X	X	X	X	X	X	X
12.b		X		X	X				X	X				X	X			X		X	X	X	X
12.c		X	X	X	X	X				X	X	X	X	X		X	X	X		X			
12.d	X	X	X	X						X		X		X		X						X	
12.e																							
13.a	X	X	X	X	X	X	X	X	X	X	X	X	X	X	X	X	X	X	X	X	X		X
13.b	X		X	X	X	X			X				X	X		X				X		X	X
13.c																							
14.a	X	X	X	X	X	X	X	X	X	X	X	X	X	X	X	X	X	X	X	X	X	X	X
14.b			X	X		X				X	X	X	X			X				X			
14.c	X	-	X	X	-	-	-	-	-	X	-	X	-	-	-	-	-	-	-	-	-	X	-
15	X	X	X	X	X	X	X	X	X	X	X	X	X	X	X	X	X	X	X	X	X	X	X
16.a	X	X	X	X	X	X	X	X	X	X	X	X	X	X	X	X	X	X	X	X	X	X	X
16.b	X	X	X	X	X	X			X	X	X	X	X	X	X	X	X	X	X	X	X	X	X
16.c																							
17	X	X		X	X	X			X	X	X	X	X	X		X	X			X	X	X	X
18	X	X	X	X	X	X	X	X	X	X	X	X	X	X	X	X	X	X	X	X	X	X	X
19			X	X	X				X	X	X	X	X	X	X	X	X	X	X	X	X	X	X
20	X	X	X	X	X	X	X	X	X	X	X	X	X	X	X	X	X	X	X	X	X	X	X
21	X	X	X	X	X	X	X		X	X	X	X	X	X	X	X	X	X	X	X	X	X	X
22										X	X		X			X			X	X	X	X	X

1.a: Indicate the study design in the title or abstract; 1.b: Summarise methods and findings in the abstract; 2: Explain the study’s background and rationale; 3: State objectives and hypotheses; 4: Present key study design elements early; 5: Describe setting, locations, and key dates; 6.a: Explain selection criteria and follow-up methods based on study type; 6.b: For matched studies, indicate criteria and number of groups; 7: Define variables, including outcomes, exposures, and confounders; 8: Detail data sources and measurement methods; 9: Explain measures to minimise bias; 10: Justify sample size; 11: Explain handling of quantitative variables; 12.a: Describe statistical methods, including confounding control; 12.b: Explain methods used for subgroup and interaction analysis; 12.c: Indicate how missing data were handled; 12.d: Explain how follow-up loss in cohort studies and matching in case–control studies were addressed; 12.e: Describe sensitivity analyses conducted; 13.a: Report participant numbers at each stage; 13.b: Explain reasons for nonparticipation; 13.c: Consider using a flow diagram; 14.a: Present participant characteristics; 14.b: Indicate missing data per variable; 14.c: Summarise follow-up time in cohort studies; 15: Report outcome data based on study type; 16.a: Present estimates with CIs; 16.b: Define categories for continuous variables if applicable; 16.c: Translate relative risks into absolute risks if relevant; 17: Report additional analyses (subgroups; sensitivity); 18: Summarise results in relation to objectives; 19: Discuss limitations and potential biases; 20: Interpret findings considering objectives, limitations, and prior evidence; 21: Discuss generalisability of results; 22: Indicate funding sources and funders’ roles. Abbreviations: A blank space indicates an absent item, an “X” indicates a present item, and a “-” indicates a non-applicable item for this type of article; STROBE, Strengthening the Reporting of Observational Studies in Epidemiology.

**Table 8:** Newcastle–Ottawa scale for risk-of-bias assessment of the included studies.

Author and Year	Study Design	Applied Scale	Total Score	Classification of Bias Risk
Filaire et al., 2001 [53]	Cohort	NOS	6/9 ★	Moderate
Teshima et al., 2002 [47]	Analytical cross-sectional studies	NOS-xs	5/9 ★	Moderate
Fleming & Costarelli, 2007 [55]	Cohort	NOS	7/9 ★	Low
Prouteau et al., 2007 [56]	Cohort	NOS	8/9 ★	Low
Ubeda et al., 2010 [37]	Analytical cross-sectional studies	NOS-xs	5/9 ★	Moderate
Zonta et al., 2011 [42]	Descriptive cross-sectional studies	NOS-xs2	3/4 ★	Low
Catikkas et al., 2013 [50]	Descriptive cross-sectional studies	NOS-xs2	2/4 ★	Moderate
Książek et al., 2014 [51]	Descriptive cross-sectional studies	NOS-xs2	1/4 ★	High
Pettersson & Berg, 2014 [36]	Analytical cross-sectional studies	NOS-xs	5/9 ★	Moderate
Reljic et al., 2015 [57]	Cohort	NOS	8/9 ★	Low
Andreato et al., 2016 [39]	Analytical cross-sectional studies	NOS-xs	6/9 ★	Moderate
Matthews & Nicholas, 2017 [54]	Cohort	NOS	5/9 ★	Moderate
Książek et al., 2017 [45]	Analytical cross-sectional studies	NOS-xs	6/9 ★	Moderate
Papadopoulou et al., 2017 [46]	Analytical cross-sectional studies	NOS-xs	7/9 ★	Low
Ribas et al., 2017 [44]	Descriptive cross-sectional studies	NOS-xs2	2/4 ★	Moderate
Anyżewska et al., 2018 [43]	Analytical cross-sectional studies	NOS-xs	8/9 ★	Low
Villarroel et al., 2018 [41]	Descriptive cross-sectional studies	NOS-xs2	3/4 ★	Low
da Luz & da Rosa, 2019 [38]	Descriptive cross-sectional studies	NOS-xs2	3/4 ★	Low
Dimitrijevic et al., 2022 [52]	Analytical cross-sectional studies	NOS-xs	5/9 ★	Moderate
Niewczas et al., 2023 [40]	Analytical cross-sectional studies	NOS-xs	7/9 ★	Low
Baranauskas et al., 2024 [48]	Analytical cross-sectional studies	NOS-xs	6/9 ★	Moderate
Liang et al., 2025 [58]	Cohort	NOS	6/9 ★	Moderate
Samanipour et al., 2025 [49]	Analytical cross-sectional studies	NOS-xs	6/9 ★	Moderate

NOS: Newcastle–Ottawa scale; NOS-xs: Newcastle–Ottawa scale suitable for analytical cross-sectional studies; NOS-xs2: Newcastle–Ottawa scale suitable for descriptive cross-sectional studies.

## Data Availability

The data presented in this study are available in the tables of this article. The PRISMA workflow data is available at https://doi.org/10.5281/zenodo.18262221, accessed on 5 March 2026.

## Supplementary Materials

The following supporting information can be downloaded at https://www.mdpi.com/article/10.3390/nu18060884/s1, Table S1: PRISMA_2020_abstract_checklist; Table S2: PRISMA_2020_checklist; Table S3: Newcastle–Ottawa scale for risk-of-bias assessment of the included cohort studies; Table S4: NOS-xs: Adaptation of the NOS for risk-of-bias assessment of the included analytical cross-sectional studies; Table S5: NOS-xs2: Adaptation of the NOS for risk-of-bias assessment of the included descriptive ross-sectional studies.

## Abbreviations

The following abbreviations are used in this manuscript:

ATP	Adenosine Triphosphate
BIA	Bioelectrical Impedance Analysis
BMD	Bone Mineral Density
BMI	Body Mass Index
DXA	Dual X-ray Absorptiometry
ISAK	International Society for the Advancement of Kinanthropometry
LEA	Low Energy Availability
MeSH	Medical Subject Headings
MMA	Mixed Martial Arts
NOS	Newcastle–Ottawa Scale
PCr	Phosphocreatine
PICO	Population/Intervention/Comparison/Outcomes
PRISMA	Preferred Reporting Items for Systematic Reviews and Meta-Analyses
STROBE	Strengthening the Reporting of Observational Studies in Epidemiology
WOS	Web Of Science

## Appendix A

**Web Of Science Search Strategy**
(“Martial arts” OR “martial arts” OR Boxing OR boxing OR karate OR kickbox-ing OR “jiu jitsu” OR “brazilian jiu jitsu” OR taekwondo OR judo OR “muay thai”) AND (“Feeding behavior” OR “feeding behav-ior” OR “dietary habit” OR “food consump-tion” OR Diet) AND (Anthropometry OR anthropometry OR “anthropometric characteristics” OR “anthropometric variables” OR “Body composition” OR “body composition” OR “Skinfold Thickness” OR skin-fold)

**WoS Advanced Search Strategy**
(ALL = “Martial arts” OR (TI = “martial arts” OR AB = “martial arts”) OR ALL = Boxing OR (TI = boxing OR AB = boxing) OR (TI = karate OR AB = karate) OR (TI = kickbox-ing OR AB = kickbox-ing) OR (TI = “jiu jitsu” OR AB = “jiu jitsu”) OR (TI = “brazilian jiu jitsu” OR AB = “brazilian jiu jitsu”) OR (TI = taekwondo OR AB = taekwondo) OR (TI = judo OR AB = judo) OR (TI = “muay thai” OR AB = “muay thai”)) AND (ALL = “Feeding behavior” OR (TI = “feeding behav-ior” OR AB = “feeding behav-ior”) OR (TI = “dietary habit” OR AB = “dietary habit”) OR (TI = “food consump-tion” OR AB = “food consump-tion”) OR ALL = Diet) AND (ALL = Anthropometry OR (TI = anthropometry OR AB = anthropometry) OR (TI = “anthropometric characteristics” OR AB = “anthropometric characteristics”) OR (TI = “anthropometric variables” OR AB = “anthropometric variables”) OR ALL = “Body composition” OR (TI = “body composition” OR AB = “body composition”) OR ALL = “Skinfold Thickness” OR (TI = skin-fold OR AB = skin-fold))

**Scopus (Basic Search) Search Strategy**
(“Martial arts” OR “martial arts” OR Boxing OR boxing OR karate OR kickbox-ing OR “jiu jitsu” OR “brazilian jiu jitsu” OR taekwondo OR judo OR “muay thai”) AND (“Feeding behavior” OR “feeding behav-ior” OR “dietary habit” OR “food consump-tion” OR Diet) AND (Anthropometry OR anthropometry OR “anthropometric characteristics” OR “anthropometric variables” OR “Body composition” OR “body composition” OR “Skinfold Thickness” OR skin-fold)

**Scopus (Advanced Search) Search Strategy**
(INDEXTERMS (“Martial arts”) OR TITLE-ABS (“martial arts”) OR INDEXTERMS (Boxing) OR TITLE-ABS (boxing) OR TITLE-ABS (karate) OR TITLE-ABS (kickbox-ing) OR TITLE-ABS (“jiu jitsu”) OR TITLE-ABS (“brazilian jiu jitsu”) OR TITLE-ABS (taekwondo) OR TITLE-ABS (judo) OR TITLE-ABS (“muay thai”)) AND (INDEXTERMS (“Feeding behavior”) OR TITLE-ABS (“feeding behav-ior”) OR TITLE-ABS (“dietary habit”) OR TITLE-ABS (“food consump-tion”) OR INDEXTERMS (Diet)) AND (INDEXTERMS (Anthropometry) OR TITLE-ABS (anthropometry) OR TITLE-ABS (“anthropometric characteristics”) OR TITLE-ABS (“anthropometric variables”) OR INDEXTERMS (“Body composition”) OR TITLE-ABS (“body composition”) OR INDEXTERMS (“Skinfold Thickness”) OR TITLE-ABS (skin-fold))
