# Supplementary material for: Body Composition and Dietary Intake of Combat Sports Athletes: A Systematic Review
Source: Nutrients. 2026 Mar 10;18(6):884. doi: 10.3390/nu18060884 (PMC13029353; doi:10.3390/nu18060884)
Supplement: Supplementary file 1 [file nutrients-18-00884-s001.zip › Supplementary Materials_JFHB.pdf]

Table S3. Newcastle-Ottawa Scale for Risk-of-Bias Assessment of the Included Cohort Studies (Scores ≥7 to 9, 4-6, <4 are Considered Low, Moderate, and High Risk, Respectively).

**Item**	**Filaire et al., 2001 [53]**	**Fleming & Costarelli, 2007 [55]**	**Prouteau et al., 2007 [56]**	**Reljic et al., 2014 [57]**	**Matthews & Nicholas, 2016 [54]**	**Liang et al., 2025 [58]**
Selection (1)		★				
Selection (2)	★	★	★	★	★	★
Selection (3)	★	★	★	★		★
Selection (4)	★	★	★	★	★	
Comparability (1)	★	★	★★	★★	★	★
Outcome (1)	★	★	★	★	★	★
Outcome (2)			★	★		★
Outcome (3)	★	★	★	★	★	★
Total Score	6/9	7/9	8/9	8/9	5/9	6/9

Selection (1): Representativeness of the exposed cohort; Selection (2): Selection of the non-exposed cohort; Selection (3): Ascertainment of exposure; Selection (4): Demonstration that outcome of interest was not present at start of study; Comparability (1): Comparability of cohorts on the basis of the design or analysis; Outcome (1): Assessment of outcome; Outcome (2): Was follow-up long enough for outcomes to occur; Outcome (3): Adequacy of follow up of cohorts. Abbreviations: A blank space indicates 0 points, "★" indicates 1 point, and "★★" indicates 2 points.

Table S4. NOS-xs: Adaptation of the NOS for Risk-of-Bias Assessment of the Included Analytical Cross-sectional Studies (Scores ≥7 to 9, 4-6, <4 are Considered Low, Moderate, and High Risk, Respectively).

**Item**	**Teshima et al., 2002 [47]**	**Úbeda et al., 2010 [37]**	**Pettersson & Berg, 2014 [36]**	**Andreato et al., 2016 [39]**	**Książek et al., 2017 [45]**	**Papadopoulou et al., 2017 [46]**	**Anyżewska et al., 2018 [43]**	**Dimitri-jevic et al., 2022 [52]**	**Niewczas et al., 2024 [40]**	**Baranauskas et al., 2024 [48]**	**Samanipour et al., 2025 [49]**
1. Representativeness of the study sample	★			★		★	★	★	★	★	★
2. Sample size						★	★		★	★	
3. Assessment of the exposure(s)	★	★	★	★★	★★	★★	★★	★	★	★	★
4. Assessment of the outcome(s)	★★	★★	★★	★★	★★	★	★★	★★	★★	★	★★
5. Adjustment for confounder(s)		★	★		★	★	★	★	★	★	★
6. Assessment of confounder(s)	★	★	★	★	★	★	★		★	★	★
Total Score	5/9	5/9	5/9	6/9	6/9	7/9	8/9	5/9	7/9	6/9	6/9

Abbreviations: A blank space indicates 0 points, "★" indicates 1 point, and "★★" indicates 2 points.

Table S5. NOS-xs2: Adaptation of the NOS for Risk-of-Bias Assessment of the Included Descriptive Cross-sectional Studies (Scores ≥3 to 4, 2, <2 are Considered Low, Moderate, and High Risk, Respectively).

Item	Zonta et al., 2011 [42]	Catikkas et al., 2013 [50]	Książek et al., 2014 [51]	Ribas et al., 2017 [44]	Villarroel et al., 2018 [41]	da Luz & da Rosa, 2019 [38]
1. Representativeness of the study sample	★	★			★	★
2. Sample size						
3. Assessment of the outcome(s)	★★	★	★	★★	★★	★★
Total Score	3/4	2/4	1/4	2/4	3/4	3/4

Abbreviations: A blank space indicates 0 points, "★" indicates 1 point, and "★★" indicates 2 points.
